# Impact of correlated noise in an energy depot model

**DOI:** 10.1038/srep19591

**Published:** 2016-01-20

**Authors:** Chunhua Zeng, Jiakui Zeng, Feng Liu, Hua Wang

**Affiliations:** 1 State Key Laboratory of Complex Nonferrous Metal Resources Clean Utilization/Faculty of Science, Kunming University of Science and Technology, Kunming 650093, P.R. China; 2 Department of Physics, Nanjing University, Nanjing 210093, P.R. China

## Abstract

Based on the depot model of the motion of active Brownian particles (ABPs), the impact of cross-correlated multiplicative and additive noises has been investigated. Using a nonlinear Langevin approach, we discuss a new mechanism for the transport of ABPs in which the energy originates from correlated noise. It is shown that the correlation between two types of noise breaks the symmetry of the potential to generate motion of the ABPs with a net velocity. The absolute maximum value of the mean velocity depends on correlated noise or multiplicative noise, whereas a monotonic decrease in the mean velocity occurs with additive noise. In the case of no correlation, the ABPs undergo pure diffusion with zero mean velocity, whereas in the case of perfect correlation, the ABPs undergo pure drift with zero diffusion. This shows that the energy stemming from correlated noise is primarily converted to kinetic energy of the intrawell motion and is eventually dissipated in drift motion. A physical explanation of the mechanisms for noise-driven transport of ABPs is derived from the effective potential of the Fokker-Planck equation.

The motion of active Brownian particles (ABPs) has been studied theoretically and experimentally because this phenomenon can explain the mechanism of self-propelled motion^1^,^2^,^3^,^4^,^5^,^6^. Self-propelled motions such as those involved in molecular motors^7^,^8^, motile bacteria^9^,^10^, migrating cells^11^, and Brownian swimmers^12^ are crucial to human life; thus, it is important to investigate the motion of microscopic biological entities, such as cells, and bacteria. For instance, on the biological level, cells or simple microorganisms are capable of active, self-driven motion, which, in several cases, has been successfully described by the Langevin or Fokker-Planck differential equations^13^,^14^,^15^,^16^. These mathematical formalisms may help to understand the dynamics of self-propelled entities^17^,^18^.

The energy depot model proposed by Schweitzer *et al.* is a major achievement in the description of self-propelled motion^19^, and the corresponding drag function was based on the idea that particles with energy^20^,^21^,^22^, such as Brownian particles with the ability to take up energy from the environment, can store their energy in an internal depot and later use this internal energy to change the environment or perform different activities, such as metabolism, motion, or signal-response behaviour^23^. This active motion has remarkable stochastic features, and noise arises from different sources that can be conveniently categorised as internal and external fluctuations^24^,^25^. Internal (additive) noise describes all of the fluctuations generated from the active nature of the system^26^. External (multiplicative) noise refers to the random variations in the damping parameters^27^,^28^; this type of noise and can act on ABPs. Historically, research on the depot model has been limited to the case of one simple source of additive noise, where the transport of ABPs originates from a force with a parabolic^19^,^20^,^21^ or linear potential^29^,^30^,^31^,^32^. However, a system is always simultaneously disturbed by both internal thermal fluctuations and external random perturbations^33^. Therefore, these investigations of the depot model may neglect key effects induced by external noise. In practice, external noise always exists and plays a significant role in dynamics^34^, such as in spatially extended systems^35^, transcriptional feedback loops^36^, yeast cell populations^37^, and so on.

We aim to simultaneously consider both internal and external fluctuations in the depot model and present a more realistic model of active motion. A natural question is whether the internal and external fluctuations are statistically correlated on the same time scale. One can imagine fluctuations arising from a common origin and thus not being independent of each other; which physically would imply that two types of noise have the same origin^38^,^39^,^40^. The microscopic realisation of correlated noise processes has been discussed^41^. Meanwhile, it appears that the correlation of internal and external fluctuations is ubiquitous in nature and often fundamentally changes the dynamics of a system^42^,^43^,^44^,^45^, such as in the cases of reentrance phenomena in a bistable kinetic model^46^, anomalous diffusion of overdamped particles^47^, multiple current reversals in a symmetrical potential^48^, photoinduced phase transitions in spin-crossover solids^49^, and resonant activation of a chemical reaction^50^. We also note that in a previous depot model proposed by Schweitzer *et al.*^19^, the effects of external noise and of the correlation between two types of noise on the mechanism of ABPs transport are ignored. Despite recent advances in active matter research, there is still a lack of theoretical foundations describing the impact of correlated noise in an energy depot model. For example, to date, no clear distinction has been made between internal and external fluctuations. It was only recently shown how noise can enhance the stability and double stochastic resonance of active Brownian motion^51^. In contrast to the case of one internal noise, the correlation between internal and external noises should be considered in the depot model. We believe that correlated noise may lead to a new mechanism for the motion of ABPs, namely, transport in which the energy stems from the correlated noise, instead of a parabolic or linear potential force.

This paper is organized as follows. First of all, the depot model of noise-driven motion is presented. Using a nonlinear Langevin approach, we derive the effective velocity potential of the Fokker-Planck equation. Second, we numerically discuss the impact of correlated noise on the transport properties of ABPs. The mechanisms for noise-driven transport of ABPs are theoretically explained by the effective potential of the Fokker-Planck equation. Finally, we summarize our results and provide concluding remarks.

## The depot model of noise-driven motion

The ABPs with an internal energy depot are given by^19^


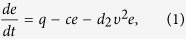


where *e* is the internal energy depot of the ABPs. Furthermore, the ABPs are able to store energy in internal energy depots, which may be altered by three different processes^20^,^23^,^29^: (i) gain of energy resulting from the environmental fluctuations induced by the noises, where *q* is the flux of energy into the depot; (ii) loss of energy by internal dissipation, which is assumed to be proportional to the internal energy. Here the rate of energy loss *c* is assumed to be constant; and (iii) conversion of internal energy into kinetic energy with a rate *d*_2_*υ*^2^, which *υ* is the actual velocity of the ABPs, and *d*_2_ > 0.0. This shows that the depot energy may be used to drive the motion of an active Brownian particle (ABP). Thus, the motion of the ABP is motivated by investigations of active biological motion, which relies on the supply of energy, which is dissipated by metabolic processes, but can be also converted into kinetic energy.

## The nonlinear Langevin equation

Let us now construct a dynamics of an ABP with unit mass under an energy depot, and subject to cross-correlated noise sources^45^,^47^. It can be described by the nonlinear Langevin equation (LE)


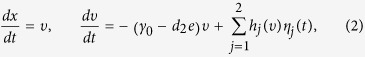


in which *x* denotes the position of the particle, *γ*_0_ is the drag coefficient of the particle at position *x*, moving with velocity *υ*. *h*_*j*_(*υ*) is deterministic function that characterize the state-dependent action of Gaussian noise *η*_*j*_(*t*), where *η*_*j*_(*t*) is Gaussian white noise and its statistical property is given by^47^


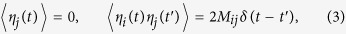


here 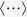 characterizes averaging with respect to the noise *η*_*j*_(*t*), *M*_11_ = *M*_1_ and *M*_22_ = *M*_2_ are the intensities of the noises *η*_1_(*t*) and *η*_2_(*t*), respectively, 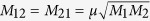, where *μ* is the intensity characterizing the cross-correlation of the noises, 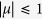. For *μ* = 0.0, two types of noise are no correlation, while for 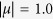, they are perfect correlation. Without loss of generality, we assume that the noise *η*_1_(*t*) is external (multiplicative) and originates in the random variations in the drag parameter^27^,^28^. We vary the drag coefficient by allowing the parameter *γ*_0_ to vary stochastically, i.e., 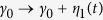. And another *η*_2_(*t*) is internal (additive) and originates from the active nature of the system. If two types of noise are simultaneously considered, we can rewrite that the motion of an ABP subject to multiplicative noise [*h*_1_(*v*) = −*v*] and additive noise [*h*_2_(*v*) = 1] as


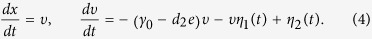


Compared to previous investigations, the transport properties of ABPs have been mainly considered for the resulting force from parabolic potential^19^,^20^ or linear potential^29^,^30^. Here, we study impact of resulting force from the cross-correlation between two noises *η*_1_(*t*) and *η*_2_(*t*) on their transport properties. Generally, an ABP obeying Eqs. (4) with (2) possesses a mean velocity of the particle 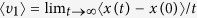, and a mean velocity of the internal energy depot 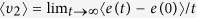, and undergoes a diffusive spread around this mean motion which is characterized by an effective diffusion coefficient 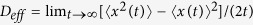.

## The effective potential of Fokker-Planck equation

To obtain the approximate Fokker-Planck equation, we will first reduce the two coupled ordinary differential equations to the state evolution equation of *υ*(*t*). Notice that *e*(*t*) can be considered to be a fast variable compared with *υ*(*t*) since 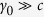, i.e., by comparison with the time scale of motion, the internal energy depot reaches fast a quasistationary equilibrium^19^. If *de*/*dt* = 0.0, we obtain


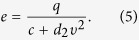


Then the fast variable *e*(*t*) from Eq. (5) is replaced in the LE (4), so the LE (4) can be rewriten as


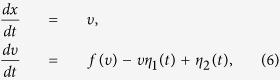


where the deterministic drag force *f*(*υ*) = −*γ*(*υ*)*υ*, and the nonlinear drag function 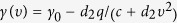. In the limit of large velocities, *γ*(*υ*) approaches the normal drag coefficient *γ*_0_, but in the limit of small velocities a negative drag occurs, as an additional source of energy for the ABPs. Hence slow particles are accelerated, while the motion of fast particles is damped^29^. The fast variable in Eq. (6) can be assumed to be at an effective equilibrium, whereas the slow variable is responsible for the dynamics of a system^19^,^20^,^29^. The deterministic velocity potential related to the deterministic drag force *f*(*υ*) in (6) is


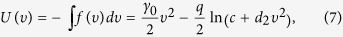


has two alternative stable states 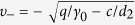, 
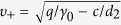
, and separated by an unstable state *υ*_*u*_ = 0.0.

Let *Q*(*υ*, *t*) denotes the velocity distribution that the velocity of the particle exactly equals *υ* at time *t*. Then, from Risken^52^, the Fokker-Planck equation of *Q*(*υ*, *t*) corresponding to Eqs. (6) with (3) can be given by


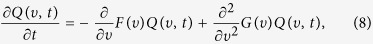


in which *F*(*υ*) and *G*(*υ*) are obtained, respectively^39^,^46^,^53^


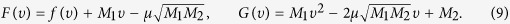


According to Eqs. (8) and (9), the stationary velocity distribution can be given by


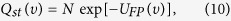


where *N* is a normalization constant, and the effective velocity potential *U*_*FP*_(*υ*) of the Fokker-Planck equation reads


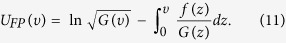


Integrating Eq. (11), we obtain


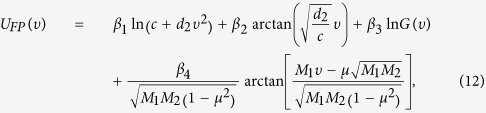


in which


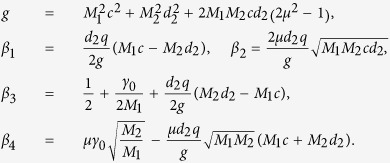


An equivalent description to the Fokker-Planck equation, which provides actual stochastic trajectories as opposed to probability distributions, is the LE. The LE corresponding to the Fokker-Planck equation (8) is^54^


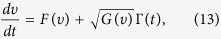


where Γ(*t*) is a Gaussian white noise with 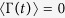, and 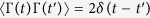. This LE (13) under the action of a noise Γ(*t*) is equal to the LE (6) under the action of two noises *η*_1_(*t*) and *η*_2_(*t*). Notice that the effective drag force in the LE (13) is 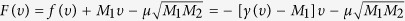. If *μ* = 0, the last term of the *F*(*υ*) vanishes and we have the contribution to the effective drag force for only the fluctuation processes (*M*_1_) of the drag coefficient, i.e., 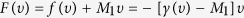. In fact, for no correlation (*μ* = 0), the additive noise (*M*_2_) has no effect on the effective drag force. Therefore, it is shown that the correlated noise (*μ* ≠ 0) can play a key role in transport properties of the ABPs, and the impacts of two types of noise *M*_1_ and *M*_2_ on them depend on the correlated noise. In addition, the noise term appears in the LE (13) with a velocity-dependent term, 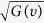, multiplying it.

## The transport properties of ABPs

Using stochastic second-order Runge-Kutta algorithm^55^,^56^,^57^, we have numerically integrated Eqs. (4) with (1, 2, 3) by a time step Δ*t* = 0.01. The initial condition is chosen randomly from a symmetric, uniform distribution over the interval [−1, 1]. The data obtained were averaged over 500 different trajectories and each trajectory evolved over 10^5^ periods.

## The mean velocity of ABPs

**Figure 1:**
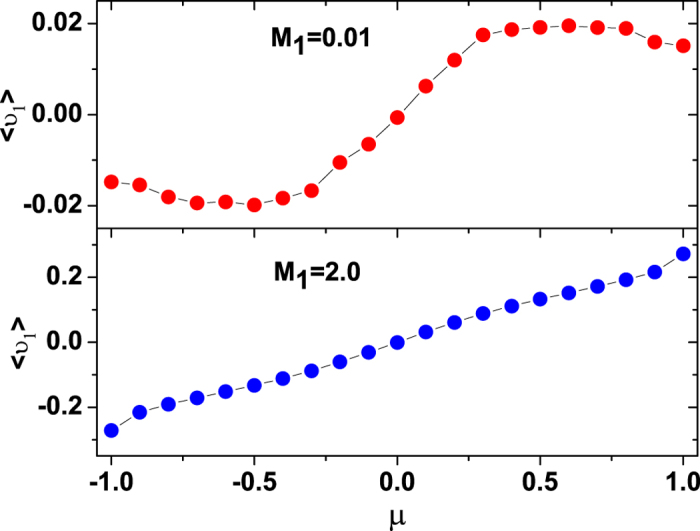
The mean velocity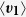 vs. *μ* for *M*_1_ = 0.01, and 2.0. The other parameters are *γ*_0_ = 20.0, *q* = 2.0, *d*_2_ = 1.0, *c* = 0.01, and *M*_2_ = 0.05.

The velocity reversal from a negative to a positive net velocity is shown in Fig. 1. Dependent on the value of the cross-correlation intensity *μ*, we see the switch from the negative to the positive value of the net velocity at a critical value of the parameter *μ* = 0.0. Because of the definition of *μ*, the results for *μ* < 0.0, are the inverse of the results for *μ* > 0.0. Obviously, for *μ* = 0.0, it is no net velocity occurs, because the two main velocities compensate. This phenomenon is noteworthy since in the absence of parabolic or linear potential, the velocity should be zero no matter what values the noise takes^17^,^19^, and different from the case in which transport depends on the bias force^18^,^29^. In our case, however, the transport reversal depends on the correlated noise. The fact that the cross-correlation between two types of noise induces a net velocity, can provide a valuable way to control the net velocity by manipulating the cross-correlation between two types of noise. For a small multiplicative noise intensity (*M*_1_ = 0.01), the absolute value of the 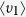 increases first and then decreases, exhibiting a maximum with the increase of 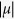, there exists one optimal value of the cross-correlation between two types of noise, in which the mean velocity takes its maximum. However for a large multiplicative noise intensity (*M*_1_ = 2.0), the absolute value of the 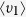 increases as 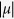 increases. That is to say, the cross-correlation between two types of noise can play opposite roles in the 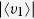 for a small multiplicative noise intensity. One is that the particle can benefit from the fluctuations induced by the cross-correlation between two types of noise, and the increase of the cross-correlation intensity can enhance the directional motion and facilitate the particle to move to the potential minimum. Another is that the strong cross-correlation between two types of noise induces the weakening influence of potential^42^,^58^, and thus leads to a decrease of the 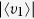. The competition of these two opposite roles leads to a maximum in the 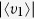 as a function of the *μ*. However for a large multiplicative noise intensity, the cross-correlation between two types of noise can just enhance the influence of the potential, and consequently an increase of the 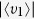.

**Figure 2:**
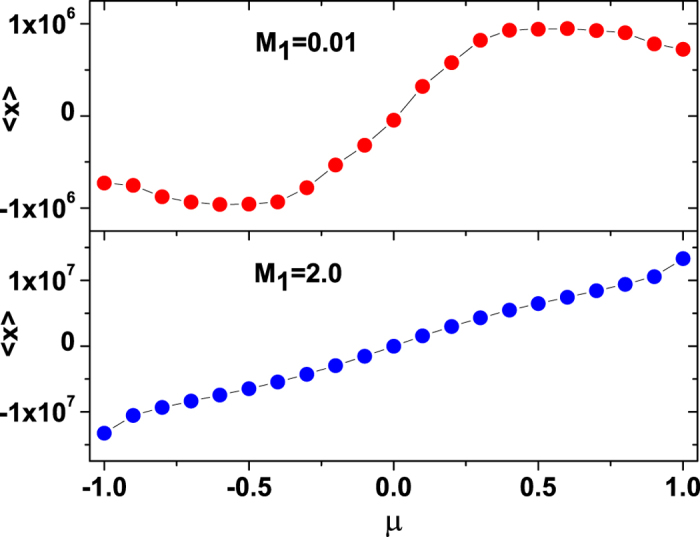
The mean position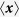 vs. *μ* for *M*_1_ = 0.01 and 2.0. The other parameters are *γ*_0_ = 20.0, *q* = 2.0, *d*_2_ = 1.0, *c* = 0.01, and *M*_2_ = 0.05.

The variation of mean position 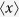 as a function of the cross-correlation intensity *μ* is shown in Fig. 2(a,b) for different values of the *M*_1_. It is shown that when two types of noise are uncorrelated (*μ* = 0.0), the 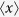 is zero, which implies the net velocity is zero. For *μ* < 0.0, the departure of the 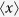 from zero towards the negative direction indicates the preferential distribution of the ABP in the 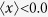. But for *μ* > 0.0, the departure of the 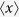 from zero towards the positive direction indicates the preferential distribution of the ABP in the 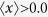. This indicates that the 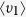 is negative for *μ* < 0.0, and positive for *μ* > 0.0, as shown in Fig. 1(a,b). For *M*_1_ = 0.01, the 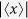 exhibits a maximum with the increase of the 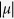. But for *M*_1_ = 2.0, the 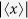 increases as the 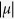 increases. It also means that the 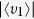 exists one optimal value of the cross-correlation between two types of noise, in which the mean velocity takes its maximum for case of *M*_1_ = 0.01, while for case of *M*_1_ = 2.0, the 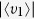 increases as the 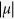 increases.

**Figure 3:**
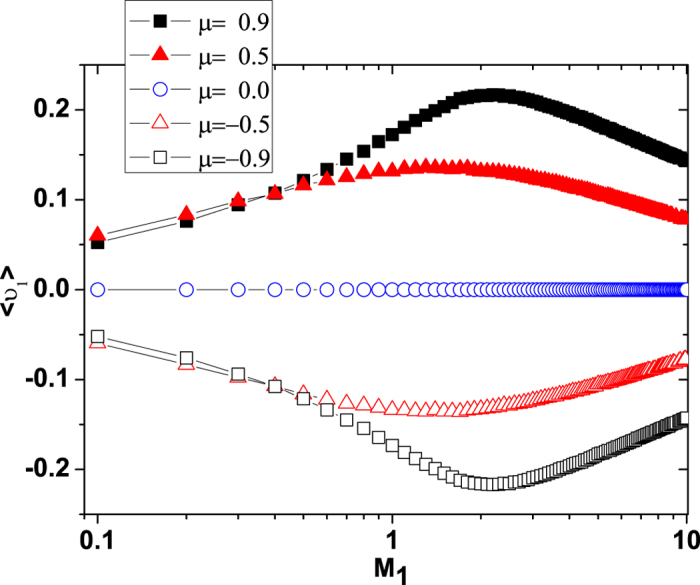
The mean velocity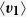 vs. *M*_1_ for *μ* = −0.9, −0.5, 0.0, 0.5 and 0.9. The other parameters are *γ*_0_ = 20.0, *q* = 2.0, *d*_2_ = 1.0, *c* = 0.01, and *M*_2_ = 0.05.

**Figure 4:**
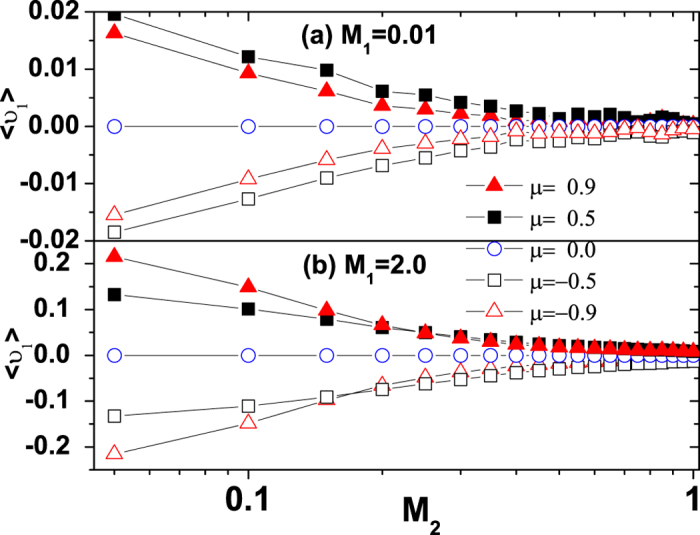
The mean velocity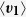 vs. *M*_2_ for *μ* = −0.9, −0.5, 0.0, 0.5 and 0.9. **(a)** *M*_1_ = 0.01; **(b)** *M*_1_ = 2.0. The other parameters are *γ*_0_ = 20.0, *q* = 2.0, *d*_2_ = 1.0, and *c* = 0.01.

The mean velocity 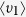 of the ABPs as functions of the multiplicative and additive noise intensities *M*_1_ and *M*_2_ is shown in Figs 3 and 4 for different values of cross-correlation intensity *μ*, respectively. It is found that 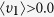 for *μ* > 0.0, 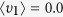 at *μ* = 0.0, and 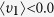 for *μ* < 0.0. In Fig. 3, the curve is observed to be bell shaped, which shows the feature of resonance, i.e., the 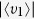 increases first and then decreases, exhibiting a maximum with the increase of the *M*_1_, there is an optimized value of the *M*_1_ in which the 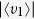 takes its maximum value. This means that a multiplicative noise intensity can facilitate the transport of ABPs. In Fig. 4, the 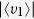 decreases as the additive noise intensity *M*_2_ increases. When *M*_2_→0.0, the 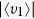 tends to zero for all values of the 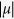. It must be pointed out from Figs 3 and 4 that for a small multiplicative noise intensity (see Fig. 3 and *M*_1_ = 0.01 in Fig. 4), the 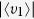 increases first and then decreases when the 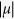 increases, but for a large multiplicative noise intensity (see Fig. 3 and *M*_1_ = 2.0 in Fig. 4), the 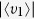 increases when the 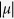 increases. These findings are also consistent with the results of Fig. 1(a,b).

**Figure 5:**
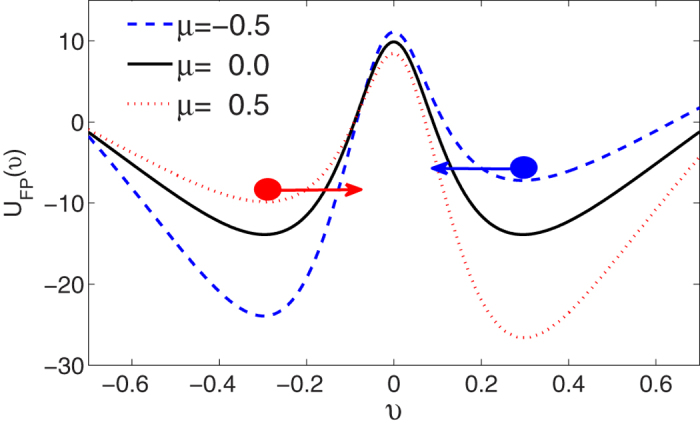
The effective velocity potential *U*_*FP*_(*υ*) vs. *υ* for *μ* = −0.5, 0.0, 0.5. The other parameters are *γ*_0_ = 20.0, *q* = 2.0, *d*_2_ = 1.0, *c* = 0.01, *M*_1_ = 0.5, and *M*_2_ = 0.05.

We provide a pictorial understanding of some of noise-driven transport of ABPs. From the physics point of view, it is well-known that the effective potential (or force) determines the transport properties of ABPs^52^. The effective potential *U*_*FP*_(*υ*) as a function of the *υ* is shown in Fig. 5 for different values of cross-correlation intensity *μ*. For *μ* = 0.0, the effective potential *U*_*FP*_(*υ*) is symmetrically distributed, thence no mean velocity of ABPs can be caused. But for *μ* ≠ 0.0, the presence of cross-correlation between two noises breaks the symmetry of potential and makes the probability of the fluctuations on the two sides of the potential barrier different, thus a net velocity of ABPs arises. The negative correlation (*μ* = −0.5) causes the potential well at *υ* = *υ*_−_ much lower than the potential well at *υ* = *υ*_+_, but the positive correlation (*μ* = 0.5) makes the potential well at *υ* = *υ*_+_ much lower than the potential well at *υ* = *υ*_−_. Therefore, a negative (or positive) correlation may be enough to displace the ball far enough to push it over the hill (potential maximum *υ*_*u*_), resulting in a shift to the alternative stable state *υ*_−_ (or *υ*_+_). Since the mean velocity of ABPs corresponds roughly to the asymmetry of the potential and the depth of the potential minimum^45^, thus the negative correlation leads to an increase in the negative velocity, while the positive correlation leads to an increase in the positive velocity(also see Fig. 1(a,b)).

**Figure 6:**
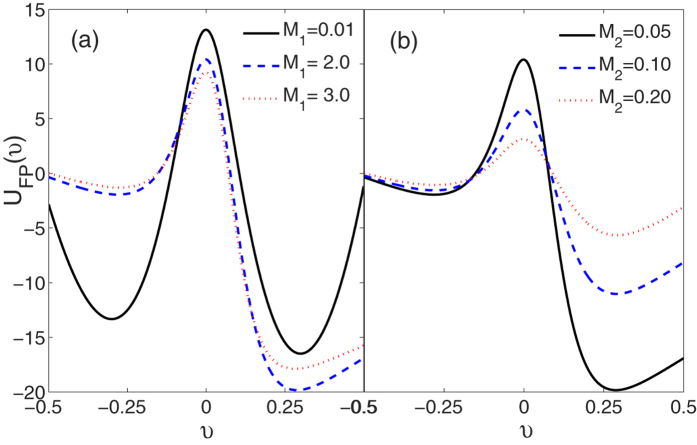
The effective velocity potential *U*_*FP*_(*υ*) vs. *υ* (**a**) *M*_2_ = 0.05, *M*_1_ = 0.01, 2.0, and 3.0; (**b**) *M*_1_ = 2.0, *M*_2_ = 0.05, 0.1, and 0.2. The other parameters are *γ*_0_ = 20.0, *q* = 2.0, *d*_2_ = 1.0, *c* = 0.01, and *μ* = 0.5.

In Fig. 6(a,b), we present that the effective potential *U*_*FP*_(*υ*) as a function of the *υ* for different values of the multiplicative noise intensity *M*_1_ and additive noise intensity *M*_2_, respectively. For *μ* = 0.5, it is found from Fig. 6(a) that the depth of potential minimum at *υ* = *υ*_+_ is increased and the asymmetry of the potential is enhanced as the *M*_1_ increases from 0.01 to 2.0. But further increasing *M*_1_ (*M*_1_ = 3.0) can also reduce the potential asymmetry in a slightly different way. This is the reason for a maximum in the 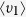 with the increase of the *M*_1_ (also see Fig. 3). From Fig. 6(b), it is seen that the minimum potential located at *υ* = *υ*_+_ is shallower and the asymmetry of the potential is also reduced as the *M*_2_ increases, which is the reason for the decrease of the mean velocity 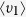 with the increase of *M*_2_ (also see Fig. 4). In short, a physical explanation of the mechanisms for noise-driven transport of ABPs is derived from the effective potential of the Fokker-Planck equation.

## The mean velocity of internal energy depot

**Figure 7:**
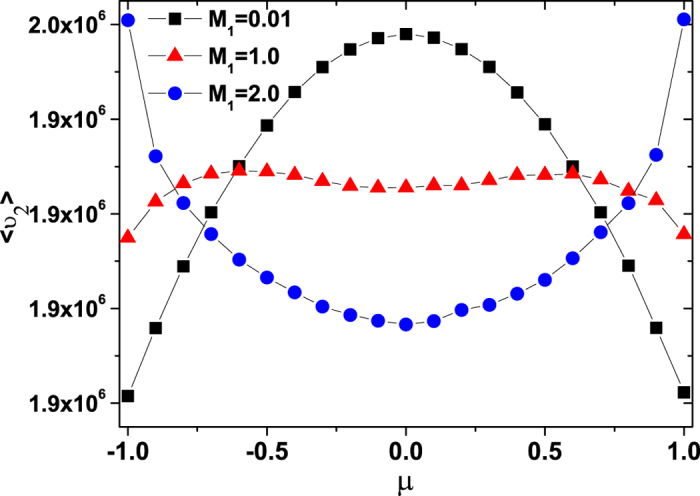
The mean velocity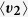 vs. *μ* for *M*_1_ = 0.01, 1.0, and 2.0. The other parameters are *γ*_0_ = 20.0, *q* = 2.0, *d*_2_ = 1.0, *c* = 0.01, and *M*_2_ = 0.05.

The mean velocity 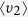 of the internal energy depot is depicted in Fig. 7 for different values of the multiplicative noise intensity *M*_1_. For small noise intensity (*M*_1_ = 0.01), the interesting point here is that there is only one peak at a value of *μ* = 0.0. However, when the value of the *M*_1_ is increased, the peak at *μ* = 0.0 vanishes and the two peaks appear at values of *μ* ≠ 0.0. As the value of the multiplicative noise intensity *M*_1_ increases continuously, the two peaks vanish and one valley appears at a value of *μ* = 0.0.

**Figure 8:**
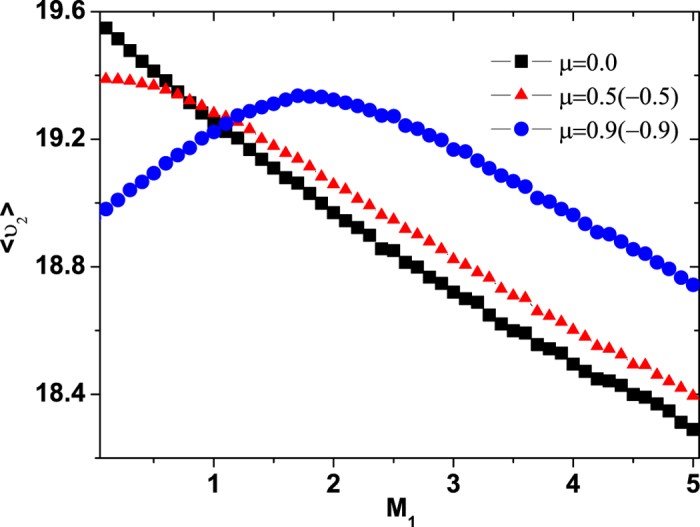
The mean velocity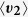 vs. *M*_1_ for 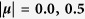 and 0.9. The other parameters are *γ*_0_ = 20.0, *q* = 2.0, *d*_2_ = 1.0, *c* = 0.01, and *M*_2_ = 0.05.

**Figure 9:**
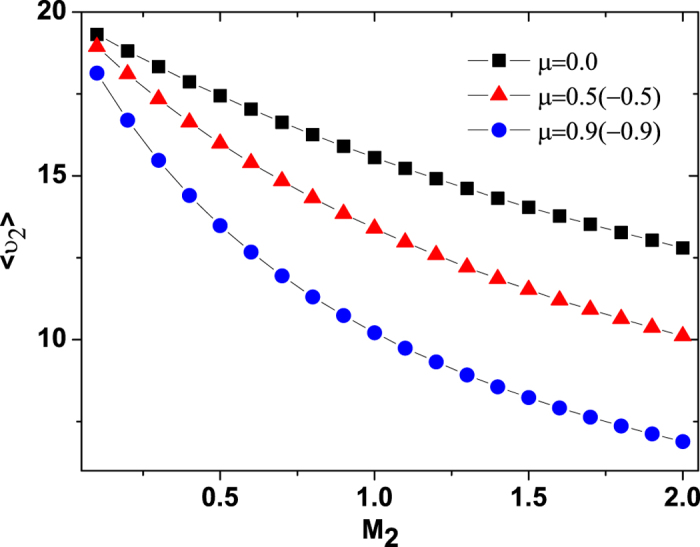
The mean velocity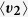 vs. *M*_2_ for 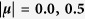 and 0.9. The other parameters are *γ*_0_ = 20.0, *q* = 2.0, *d*_2_ = 1.0, *c* = 0.01, and *M*_1_ = 0.01.

In Figs 8 and 9, we present the 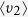 as functions of the multiplicative and additive noise intensities *M*_1_ and *M*_2_ for different values of cross-correlation intensity *μ*, respectively. From Fig. 8, it is found that for small cross-correlation intensity *μ*, the 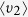 decreases as the multiplicative noise intensity *M*_1_ increases. But for large cross-correlation *μ*, there appears a maximum value of the 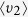 at 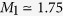 as the *M*_1_ increases. Furthermore, for small multiplicative noise intensity *M*_1_, the 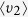 decreases as the cross-correlation intensity *μ* increases, while for large multiplicative noise intensity *M*_1_, the 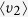 increases as the cross-correlation intensity *μ* increases. From Fig. 9, it is found that the 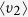 always decreases as the additive noise intensity *M*_2_ or the cross-correlation intensity *μ* increases.

Further support for this mechanism comes from the energy depot of the active motion, the ABPs have the ability to take up energy from the environmental fluctuations, to store it in an internal depot, and to convert internal energy into kinetic energy^19^,^20^,^23^,^29^. It is found from Figs 7 and 8 that there exists an optimal value of the multiplicative noise intensity *M*_1_ or the cross-correlation intensity *μ* at which the 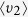 of the internal energy depot is maximised. However, the 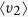 decreases monotonically as the additive noise intensity *M*_2_ increases (see Fig. 9). This is also for the reason that a multiplicative noise or a cross-correlation can facilitate the transport of the ABPs in which the its mean velocity of the ABPs takes a maximum (see Figs 1 and 3), and the mean velocity of the ABPs tends to zero with the increase of additive noise intensity(see Fig. 4). Therefore, facilitated transport of ABPs can be induced by multiplicative noise or by the cross-correlation between two types of noise, and the multiplicative noise intensity or cross-correlation intensity can be used as a valuable parameter for controlling the internal energy depot.

## The effective diffusion of ABPs

**Figure 10:**
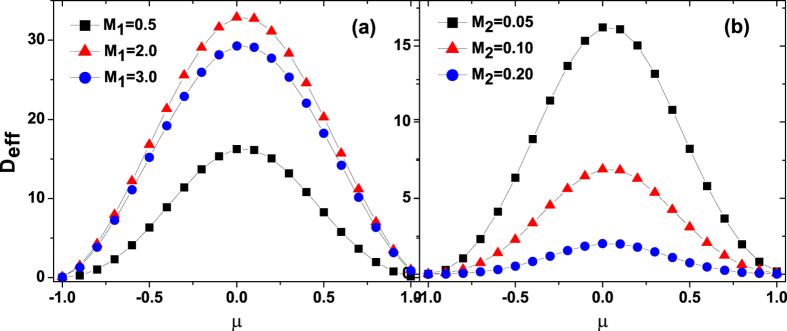
The diffusion coefficient *D*_*eff*_ vs. *μ*. (**a**) *M*_2_ = 0.05, *M*_1_ = 0.5, 2.0, and 3.0; (**b**) *M*_1_ = 0.5, *M*_2_ = 0.05, 0.1, and 0.2. The other parameters are *γ*_0_ = 20.0, *q* = 2.0, *d*_2_ = 1.0, and *c* = 0.01.

Figure 10 displays the effective diffusion *D*_*eff*_ as a function of the cross-correlation intensity *μ* for different values of the multiplicative noise intensity *M*_1_ and the additive noise intensity *M*_2_, respectively. It is shown that the effective diffusion increases first and then decreases, exhibiting a maximum at *μ* = 0.0 with the increase of *μ* from −1.0 to 1.0. On the one hand, the effective diffusion is stronger in the *μ* = 0.0 (symmetric) case than in the *μ* ≠ 0.0 (asymmetric) case, which is most pronounced when the underlying potential is symmetric. On the other hand, in the case of *μ* = 0.0 (no correlation), the ABPs perform pure diffusion with 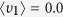, whereas in the case of 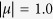 (perfect correlation), the ABPs perform pure drift with *D*_*eff*_ = 0.0. The reason for suppressing the diffusion means that the cross-correlation between two types of noise breaks the symmetry of the potential to generate motion of the ABPs with a net velocity, i.e., the diffusion is suppressed because the energy stemming from the correlated noises is primarily converted to kinetic energy of the intrawell motion and finally dissipated in the drift motion. In addition, it is shown from Fig. 10(a) that the effective diffusion increases first and then decreases as the multiplicative noise intensity *M*_1_ increases. However from Fig. 10(b), it is found that the effective diffusion always decreases as the additive noise intensity *M*_2_ increases.

## Concluding remarks

In this paper, we have studied the impact of correlated noise on the transport properties of ABPs in an energy depot model. Using a nonlinear Langevin approach, we demonstrate a new mechanism for the transport of ABPs, in which the energy stems from the correlated noise. The correlation between two types of noise breaks the symmetry of the potential to generate motion of the ABPs with a net velocity. This is different from the case in which transport depends on the bias force^18^,^29^. The absolute maximum value of the mean velocity of ABPs depends on the correlated noise or the multiplicative noise, whereas a monotonic decrease in the mean velocity occurs with additive noise. Further support for this mechanism is obtained from the energy depot of the active motion^19^,^20^. It is found that there exists an optimal multiplicative noise intensity or cross-correlation intensity at which the mean velocity of the internal energy depot is maximised; however, the velocity decreases monotonically as the additive noise intensity increases. This phenomenon occurs because a multiplicative noise or a cross-correlation can facilitate the ABP transport when the mean velocity of the ABPs is maximised, while a monotonic decrease is observed with additive noise. Therefore, the ABP transport can be induced by multiplicative noise or by cross-correlation between two types of noise, and the multiplicative noise intensity or cross-correlation intensity can be used as a valuable parameter for controlling the internal energy depot. When there is no correlation, the ABPs undergo pure diffusion with zero mean velocity, but when there is perfect correlation, the ABPs undergo pure drift with zero diffusion. The diffusion is suppressed because the energy stemming from the correlated noises is primarily converted to kinetic energy of the intrawell motion and finally dissipated in the drift motion. A physical explanation of the mechanisms for noise-driven transport of ABPs is derived from the effective potential of the Fokker-Planck equation. Our findings may be helpful in understanding the active (self-propelled) motion of biological processes, especially in understanding the single cell motility and intracellular transport that appears in various biological contexts, both within cells and on the multicellular level.

## Additional Information

**How to cite this article**: Zeng, C. *et al.* Impact of correlated noise in an energy depot model. *Sci. Rep.* **6**, 19591; doi: 10.1038/srep19591 (2016).
